# Roles of STAT3 in the pathogenesis and treatment of glioblastoma

**DOI:** 10.3389/fcell.2023.1098482

**Published:** 2023-02-27

**Authors:** Weijia Fu, Xue Hou, Lihua Dong, Wei Hou

**Affiliations:** ^1^ Department of Radiation Oncology & Therapy, The First Hospital of Jilin University, Changchun, China; ^2^ Jilin Provincial Key Laboratory of Radiation Oncology & Therapy, The First Hospital of Jilin University, Changchun, China; ^3^ NHC Key Laboratory of Radiobiology, School of Public Health, Jilin University, Changchun, China

**Keywords:** stat3, glioblastoma, radiotherapy, targeted therapy, combination therapy

## Abstract

Glioblastoma (GBM) is the most malignant of astrocytomas mainly involving the cerebral hemispheres and the cerebral cortex. It is one of the fatal and refractory solid tumors, with a 5-year survival rate of merely 5% among the adults. IL6/JAK/STAT3 is an important signaling pathway involved in the pathogenesis and progression of GBM. The expression of STAT3 in GBM tissues is substantially higher than that of normal brain cells. The abnormal activation of STAT3 renders the tumor microenvironment of GBM immunosuppression. Besides, blocking the STAT3 pathway can effectively inhibit the growth and metastasis of GBM. On this basis, inhibition of STAT3 may be a new therapeutic approach for GBM, and the combination of STAT3 targeted therapy and conventional therapies may improve the current status of GBM treatment. This review summarized the roles of STAT3 in the pathogenesis of GBM and the feasibility of STAT3 for GBM target therapy.

## 1 Introduction

Glioblastoma (GBM) is the most malignant type of glioma featured by fast proliferation, strong invasion, and poor prognosis (Peng et al., 2018). Patients with GBM usually show a variety of clinical manifestations that are caused by dysfunction of affected areas of the brain, such as lethargy, apathy, blindness, seizures, and language changes (McKinnon et al., 2021). Besides, it often grows in the brain parenchyma in an invasive manner (Lah et al., 2020), which subsequently invades the blood vessels and nerves, resulting in tumor migration to the central nervous system (CNS) (Louis et al., 2016).

*STAT3* gene is a family member of *STAT* genes encoding the STAT protein that can be activated by a variety of cytokine receptors, which then mediates massive biological processes and pathogenesis of several malignancies such as GMB. *STAT3* gene is localized on chromosome 17 of the human genome, with similar structure to the other STAT proteins. STAT3 protein consists of a conserved amino acid terminal, DNA ligand, a SH3 domain, a SH2 domain, and a transcriptional activation C-terminal domain (Yu et al., 2014).

Accumulating evidence indicates that JAK/STAT3 pathway plays a vital role in the pathogenesis of several malignancies. As the upstream element of JAK/STAT3 signaling pathway, IL-6 is reported to play crucial roles in the activation of JAK/STAT3 pathway. First, the IL-6 could bind the glycoprotein 80 (GP80) that served as the non-signal transduction component of IL-6 receptor. Then the IL-6-GP80 complex could bind the GP130 that served as the signal transduction component of IL-6 receptor, which then formed the IL6-GP80-GP130-JAK complex (Johnson et al., 2018). The binding of intracellular portion of GP130 would lead to tyrosine phosphorylation of JAK, and the activated JAK would trigger the phosphorylation of tyrosine residues on another intracellular segment of GP130 by releasing phosphate. These phosphorylated tyrosine sites functioned as the harboring sites for the surrounding amino acid sequences, thereby recruiting the transcriptional factor STAT to the SH2 structural domain. Subsequently, the tyrosine in STAT was activated upon the activation of JAK. After separation from the receptor, its nuclear localization signal was exposed followed by entering into the nucleus, which triggered the gene transcription by binding to the target gene. Thus, STAT3 signaling pathway was crucial for the pathogenesis of several malignancies (Stahl et al., 1994; Garbers et al., 2018). However, their roles in the pathogenesis of GBM are still not well defined. This review aimed to summarize the roles of STAT3 activation in pathogenesis and progression of GBM and discuss the potency of STAT3 inhibitors for GBM therapy.

## 2 Relationship between STAT3 and GBM

### 2.1 STAT3 expression in patients with different glioma grades

STAT3 expression varies in patients with different glioma grades. Studies have shown that p-STAT3 expression was not expressed in normal brain tissue specimens or patients with WHO grade II low-grade astrocytoma (LGA). Among 17 patients with WHO grade III anaplastic astrocytomas (AA) expression of p-STAT3 was detected in 9 (53%) patients. In 60 patients with WHO grade IV GBM or gliosarcoma, p-STAT3 expression was detected in 32 (53%) patients. In contrast, in oligodendrogliomas or mixed oligoastrocytomas (MOA), increased p-STAT3 expression showed no correlation with increased tumor grade. For example, p-STAT3 expression was detected in 38% of oligodendroglioma patients (n = 16), 40% of anaplastic oligodendroglioma (AO) patients (n = 15), and 100% of WHO grade II MOA patients (n = 6). Whereas, it was merely expressed in 58% of WHO grade III A MOAs patients. All these indicated that the p-STAT3 expression showed no correlation with the tumor grade of oligodendroglioma or MOA tumor grade (Abou-Ghazal et al., 2008).

### 2.2 Kinases involved in STAT3 phosphorylation/regulation in GBM pathogenesis

Several factors have been reported to be associated with the activation of STAT3 in GBM, including positive regulator (e.g., BMX), and downstream cytokines of the STAT3 signaling pathway [e.g., growth factor receptor (EGFR), platelet growth factor (PDGF), c-Met, and Suppressor of cytokine signaling (SOCS)]. For example, *EGFR* amplification was identified in about 60% of GBM cells, resulting in imbalance in the STAT3 signal pathway (Zadeh et al., 2013). SOCS3, a member of the SOCS family centered on the SH2 structural domain, is one of the target genes of STAT3. Consequently, SOCS3 can inhibit the phosphorylation of STAT3, resulting in a negative feedback regulatory loop. In approximately 20%–30% of GBM cells, there was a high hypermethylation in the *SOCS* promoter, while some regulatory factors were highly expressed in GBM stem cells. They can bind to SOCS3 (e.g., MiR-30) and then inhibit the downstream pathways, leading to inactivation of the *SOCS* gene and consequent reduction on the inhibitory effects on STAT3. Then the inactivation of *SOCS3* gene will further trigger activation of EGFR-related signaling pathways, which leads to aberrant activation of STAT3 in GBM (Zhou et al., 2017). Therefore, over-expression of EGFR and inhibition of the SOCS family were closely related to the poor prognosis among GBM patients (Lindemann et al., 2011). Bone marrow kinase x (BMX), a member of the Tec family, was expressed in renal cell carcinoma, prostate cancer, breast cancer, and other malignancies (Uckun and Venkatachalam, 2021). Upregulation of BMX kinase was reported in the malignant GBM stem cells. Also, it could activate the STAT3 in GBM cells without affecting normal neural stem cells. In the presence of *BMX* gene knockdown, the activation of STAT3 was strongly inhibited and the expression of GSC transcription factor was significantly suppressed (Guryanova et al., 2011). This indicated that BMX involved in the maintenance of unlimited self-renewal capacity of GBM stem cells by activating STAT3 signaling pathway.

The positive rate of p-STAT3 in human GBM was up to 60%, and was closely related to the histological grade, invasion and metastasis, as well as poor prognosis (Lindemann et al., 2011). In addition, several factors contributed to the STAT3 phosphorylation, including ribosomal protein L34 (RPL34), protein inhibitor of active STAT3 (PIAS3), and trim sequence protein (TRIM) family SH3GL2 (Saini et al., 2018; Ji et al., 2019; Pan et al., 2019). STAT3 phosphorylation was accomplished by a tyrosine residue (Tyr705) located in the SH2 structural domain and a serine phosphorylation site at residue 727 (Ser727) within the C-terminal structural domain. Phosphorylation of Tyr705 is the most common of STAT3 modifications and is generally mediated by JAK kinases recruited to the cytoplasmic tail of the receptor. Phosphorylated Tyr705 can further promote the phosphorylation of Ser727, which in turn promote the phosphorylation of Y705. Their interaction ultimately leads to the phosphorylation of STAT3, prompting STAT3 activation of target genes (Giannopoulou et al., 2022). Furthermore, phosphorylation and nuclear translocation of STAT3 at protein Tyr705 showed increase in the presence of radiation, in a dose- and time-dependent manner. This was possibly associated with the radiation-induced EGFR activation or IL-6 secretion in GBM cells (Chautard et al., 2010).

STAT3 may promote invasion by upregulating proinvasive factors such as matrix metalloproteinase-2 (MMP2), MMP -9, and adherent spot kinase (FAK) (Park et al., 2017). STAT3/miR-182-5p/tumor suppressor protein-8 (PCDH8) signaling also promotes the migration and invasion of glioma cells (Xue et al., 2016). Finally, STAT3 may also play a role in the induction of a more aggressive phenotype by interacting with hypoxia-inducible factor 1 (HIF-1) and vascular endothelial growth factor (VEGF) (Xu et al., 2005). Notch pathway has been reported to associate with STAT3 activation as it was shown that Delta-like 4 (DLL4) and Jagge1 (Jag1) Notch ligands were activated through STAT3 serine 727 phosphorylation to promote human embryonic stem cell survival. Such Notch-mediated effect on normal stem cell can be blocked by the Notch pathway inhibitor DAPT, a gamma-responsive enzyme inhibitor that impairs progerin 1 activation of Notch cleavage. Furthermore, Fan et al. demonstrated that blocking the activated Notch pathway in GBM stem cells reversed the accumulation of pSTAT3-S727 with gamma-secretase inhibitor 18 (GSI-18), which in turn selectively hindered cell proliferation. This implied that Notch played dominant roles in activating STAT3-S727. These findings suggested the existence of a regulatory loop involved in the activation of STAT3 and Notch signaling, as the Notch pathway regulated PSTAT3-S727, while PSTAT3Y705 is involved in regulating activated NOTCH signaling in glioma stem cells together with NF-κB (Zhang et al., 2020).

In addition to increased upstream activator activity, any loss-of-function mutation or decreased upstream repressor activity may explain the structural activation of STAT3 in gliomas. Examples include the STAT3 negative regulators PIAS3 and PTPRD (Brantley et al., 2008; Ortiz et al., 2014). Consistent with the overexpression of PSTAT3-Y705 and PSTAT3-S727, PIAS3 expression was lower in GBM tissues than that in non-tumorigenic brain tissues. Furthermore, inhibition of PIAS3 by RNA interference in the U87-MG human glioma cell line promoted cell proliferation despite less or no change in STAT3 phosphorylation. In contrast, overexpression of PIAS3 in the U251-MG human glioma cell line inhibited the expression of the OSM-enhanced STAT3 target genes (e.g., *Survivin*, *Bclxl*, and *SOCS3*), which resulted in reduction of cell proliferation by 80%.

PTPRD belongs to a family of protein tyrosine phosphatases that are involved in the regulation of many normal and cancer cell processes such as adhesion, proliferation and migration by regulating multiple cellular signaling pathways. PTPRD function is frequently inactivated by genetic and epigenetic alterations in GBM and other cancers, and is associated with poorer patient prognosis (Veeriah et al., 2009). A recently published functional analysis in mice by Ortiz et al. showed that PTPRD heterozygosity deletion leads to PSTAT3-Y705 accumulation and promotes glioma development in concert with CDKN2A/p16IN4KA (Ortiz et al., 2014).

### 2.3 Roles of STAT3 activation and inhibition in GBM

STAT3 is necessary for the proliferation and maintenance of pluripotency of GBM stem cells (Sherry et al., 2009). Both STAT3 and p-STAT3 were highly expressed in human GBM tissues, but were low or even rarely expressed in normal brain tissues (Li et al., 2018). In GBM stem cell lines (e.g., GS6-22 and GS7-2), there was phosphorylation of Tyr705 and Ser727 followed by stimulating of STAT3 activation. This demonstrated the aberrant activation of STAT3 in glioma stem cells (Sasse et al., 1997; De Vos et al., 2000). STAT3 inhibitors could inhibit the expression of the downstream genes by targeting the SH2 domain of STAT3, thereby preventing the binding of STAT3 dimer to DNA (Song et al., 2005; Siddiquee et al., 2007). These inhibitors could reduce STAT3-DNA binding in GBM cells using radio-labeled SIE probes with a high STAT3 specificity (Sherry et al., 2009). Moreover, they can inhibit the formation of neurosphere in GBM stem cells. Specifically, the formation of neurospheres in GBM stem cells in a medium with STAT3 small molecule inhibitors was significantly reduced compared to the control group treated with dimethyl sulfoxide (DMSO) (Liu et al., 2010). The permeability of 5-ethynyl-2′-deoxyuridine (EdU) in the GBM cells treated with STAT3 inhibitor was much lower than that in the DMSO-treated cells. This indicated that inhibition of neurosphere formation mediated by STAT3 inhibitor was associated with the decreased cellular proliferation. Meanwhile, infected GBM cells with knockdown of *STAT3* gene hindered neuroglobin synthesis, which implied that STAT3 suppression inhibited the proliferation of GBM cells (Li et al., 2010).

GBM cells treated with specific STAT3 inhibitor JSI-124 showed decrease in cellular density, together with obvious morphological changes such as cell deformation and shorter tapering processes (Su et al., 2008). Meanwhile, the proportion of GBM cells in G1 phase treated with STAT3 siRNA was significantly higher than that of the control group, which implied that inhibition of STAT3 triggered the accumulation of GBM cells arrested in the G1 phase. Moreover, STAT3 silencing contributed to significant decrease of cyclin D1 that played crucial roles in the cell division from G1 phase to the S phase (McFarland et al., 2013). These results suggested that G1 phase arrest of GBM cells induced by STAT3 may be related to the downregulation of cyclin D1 expression (Li et al., 2009).

Similarly, STAT3 inhibitors can significantly inhibit the growth and differentiation of GBM cells under *in vitro* conditions, but the inhibitory effects of STAT3 inhibitors on tumors under *in vivo* conditions were not pronounced (Han et al., 2019). Presumably, this may be related to the fact that there are many other cells and cytokines in the tumor microenvironment *in vivo*.

### 2.4 STAT3-mediated effects in epithelial mesenchymal transition (EMT)

EMT refers to a physiological process in which epithelial cells acquire the motile and invasive characteristics of mesenchymal cells. During EMT onset, the ability of intercellular tight junctions and adhesions is reduced and their ability to migrate at will is enhanced, which is characterized by upregulation of mesenchymal markers (e.g., waveform proteins and N-calmodulin) and downregulation of the expression of epithelial markers (e.g., keratin and E-calmodulin) in general epithelial cells (Nieto et al., 2016; Zhang et al., 2018b). IL-6/JAK2/STAT3 activation is mediated by the upregulation of EMT-induced transcription factors (e.g., Snail Zeb1, JUNB and Twist-1) to induce EMT, together with enhancing tumor cell migration and motility by activating the adherent patch kinase (FAK) (Jin, 2020). Recent studies have identified STAT3 and CCAAT enhancer binding protein β (C/EBPβ) as co-initiators and major regulators of GBM mesenchymal transition. Recently, the RTVP-1 gene referring as a p53-acting target gene containing a putative signal peptide, a transmembrane structural domain and an SCP structural domain has been reported to be highly expressed in mesenchymal GBM subtypes. In addition, its expression was associated with the expression of STAT3 and C/EBPβ, as C/EBPβ and STAT3 can bind to the RTVP-1 promoter. RTVP-1 overexpression facilitated glioma cell proliferation, invasion, and anchorage-independent growth, while its silencing induced the apoptosis of glioma cells. Furthermore, IL-6 treated glioma cells could upregulate the expression of RTVP-1 and enhance the RTVP-1 promoter activity by activating STAT3. In contrast, IL-6 silencing abrogated the effects of RTVP-1 on glioma cell migration and expression of the mesenchymal markers (e.g., fibronectin and α-SMA) (Giladi et al., 2015).

### 2.5 Roles of STAT3 in GBM microenvironment

STAT3 activation was reported to inhibit the activity of cytotoxic T lymphocytes and natural killer (NK) cells, as well as the maturation of dendritic cells (DCs) (Kitamura et al., 2017). Meanwhile, a variety of immunosuppressive cells such as M2 tumor-related macrophages, myeloid inhibitory cells, and regulatory T cells were recruited by STAT3, thereby inhibiting the immune response. Then more immunosuppressive molecules were released from the immune environment, together with recruitment of more immunosuppressive cells, which then formed a vicious circle of the immunosuppressive GBM microenvironment (Owen et al., 2019; Zou et al., 2020). In addition, long-term chronic inflammation promoted tumorigenesis, while STAT3 played a key role in the selective induction and maintenance of the inflammatory microenvironment during the initiation of malignant transformation and tumor progression (Loh et al., 2019), which can promote the emergence and growth of GBM cells by mediating extracellular signals of inflammatory mediators. When cells were stimulated by external signals, STAT3 was then stimulated by JAK2, MAPK, or mTOR kinases. As the STAT3 in the cytoplasm can be dimerized and activated by its phosphorylation at Y705 and S727 (Peron et al., 2021), the activated STAT3 was then translocated into the nucleus and bind to genomic DNA, exerting a regulatory role in the transcription.

In unstimulated cells, STAT was generally in an inactivated state, and inflammatory factors from IL-6 family members (e.g., IL-6, IL-11, OSM, and LIF) can rapidly activate the downstream STAT3 signaling pathway through their receptor-coupled protein. A large amount of IL-6 existed in the GBM microenvironment, which could activate STAT3 through the above inflammatory pathways and stimulate the growth and migration of GBM cells (Yu et al., 2009). The progeny generated from GBM cells in the absence of IL-6, was homogeneous, demonstrating that IL-6 contributed to increased GBM heterogeneity and tumor formation (Inda et al., 2010). Besides the IL-6 family members, inflammatory factors such as IL-10, IL-18, IL-21, IL-23, and IL-27 could also directly promote the activation of STAT3 by binding to receptors on the cell surface.

Extrinsic players in the tumor microenvironment, particularly tumor stromal cells that interact closely with cancer cells, also contribute to the therapeutic resistance in GBM. Deciphering, disrupting and exploiting the tumor microenvironment has become the forefront of anticancer research. The interstitial environment of GBM consists of multiple components, including endothelial cells, astrocytes, and some non-cellular components, as well as immune cells. Among them, astrocytes are the main component of the CNS, which are the most numerous non-neural cells in the human brain, with about 5.0-fold higher than the other neurons (Verdonck et al., 1987). Astrocytes involve in several physiological processes, such as structural and metabolic support to neurons, regulation on synaptic activity and extracellular ion distribution, as well as maintenance of the blood-brain barrier. GBM-associated astrocytes promoted the survival, proliferation, migration, invasion, and resistance to tumor cell destruction by foreign drugs of GBM cells (Mega et al., 2020). In a co-culture system with astrocytes, glioma cells upregulated the expression of STAT3 target molecules (e.g., cell cycle protein D1, MMP2 and Bcl-2) that regulated anti-apoptosis, proliferation, and motility. This was dependent on astrocyte-derived IL-6 and was reversed in the presence of IL-6 knockdown in astrocytes. It was shown that there was a cross-activation of IL6/STAT3 between glioma cells and astrocytes. Glioma cell-derived IL-6 activated STAT3, which upregulated IL-6 expression in astrocytes. Subsequently, astrocyte-derived IL-6 acted on glioma cells, leading to further STAT3 activation. This would enhance downstream events and promote glioma cell proliferation, migration, invasion and anti-apoptosis. The mutual activation between glioma cells can be inhibited by a nanomedicine (i.e., Nano-DOX), which inhibits STAT3 activation in glioma cells, thereby abolishing IL-6-mediated STAT3 cross-activation in astrocytes and its promotion of glioma cells. In Nano-DOX-BMDM-treated mice, Nano-DOX can be delivered to GBM *via* GBM-associated immune cells (e.g., macrophages) to inhibit STAT3 activation in glioma cells and reduce their export of IL-6 to astrocytes, thereby abolishing feedback activation of astrocytes to glioma cells (Chen et al., 2020b).

### 2.6 Effects of STAT3 activation on myeloid derived suppressor cells (MDSCs)

MDSCs are a highly heterogeneous class of myeloid-derived cells that develop as one of the major components of the immunosuppressive network (Gabrilovich, 2017). With T cells as the primary target, MDSCs showed immunosuppressive effects through a variety of cytokines including arginase1 (ARG1), ROS, inducible nitric oxide synthase (iNOS), NO, TGFβ, IL-10, COX2, indoleamine 2,3-dioxygenase (IDO), cysteine sequestration, and reduced T cell expression of L-selectin (Kumar et al., 2016b).

MDSCs are comprised of two major subsets, including monocytic MDSCs (M-MDSCs) and polymorphonuclear MDSCs (PMN-MDSCs). PMN-MDSCs share phenotypic and morphological features with neutrophils. Morphological features are similar to neutrophils. Whereas, M-MDSCs are similar to monocytes (Tcyganov et al., 2018). The immunosuppressive roles of M-MDSCs are closely related to NO and cytokine production, with which to suppress T-cell responses in an antigen-specific and non-specific manner, while that of the PMN-MDSC is mainly in an antigen-specific manner. Specifically, the PMN-MDSCs could mediate the antigen-specific T-cell tolerance by taking up soluble antigens and presenting them to CD8^+^ T cells in the presence of MHC class I (Gabrilovich et al., 2012). In addition to immunosuppressive effects, MDSCs influence the remodeling of the tumor microenvironment and tumor angiogenesis through inducing the production of VEGF, bFGF, Bv8, and MMP9, thereby promoting tumor progression (Shojaei et al., 2009; Tartour et al., 2011).

STAT3 promotes MDSC aggregation by inhibiting the terminal differentiation of immature myeloid cells. It has been shown that hypoxic conditions in the tumor microenvironment induce upregulation of HIF-1 and promote differentiation of MDSCs to M-MDSCs. M-MDSCs can upregulate their STAT3 expression, which then prevented their differentiation into macrophages or DCs (Corzo et al., 2010). Upon migration to the tumor microenvironment, M-MDSCs upregulate the activity of CD45 tyrosine phosphatase, which then lead to activation of STAT3. After migrating to the tumor microenvironment, M-MDSCs activate the CD45 tyrosine phosphatase, which selectively inhibit the activity of STAT3. Subsequently, the cells differentiate into TAM and M2-like TAM, which are closely related to tumor pathogenesis (Kumar et al., 2016a).

Compared to healthy control, the expression of M-MDSC and PMN-MDSC was significantly higher in the blood of GBM patients. On the contrary, MDSC in tumor tissue consisted exclusively of CD15^+^ PMN-MDSC (Gielen et al., 2015). In GBM tissues, most of the CD15^+^ cells were distributed around blood arteries, with CD15^+^ cells mostly distributed in the periarterial area. This indicated that MDSC can cross the blood-brain barrier and infiltrate GBM tissues (Moyes et al., 2018). Through myeloid cell-specific upregulation of apoptosis inhibitor 6 (API6) or autocrine IL-6, MDSC can activate STAT3 and facilitate the immune escape of GBM cells (Del Bianco et al., 2021).

### 2.7 Effect of STAT3 activation on DCs differentiation and function

As antigen-presenting cells (APCs), DCs are the cornerstone of the human immune system and can activate tumor-specific T-cell responses (Jhunjhunwala et al., 2021). There is aberrant DC differentiation and activation in the GBM microenvironment when IL-6 and IL-10 are induced, which can induce immunological tolerance in CD8^+^T cells (Bauer et al., 2009). STAT3 is activated in tumor cells and different immune cells in the GBM microenvironment, leading to severe immunosuppression (Chen et al., 2020a). Aberrant activation of STAT3 in GBM cells led to IL-10 production and DCs maturation inhibition (Assi et al., 2014). In cellular experiments, JSI-124 or siRNA targeting STAT3 silencing greatly inhibited the growth of several human and mouse GBM cell lines, which promoted the conversion of immature DCs to mature DCs, and facilitated to the recruitment of mature DCs (Turkson and Jove, 2000; Wang et al., 2004). In addition, the IL-6-STAT3 inflammatory axis inhibited the maturation of LPS-induced DCs by enhancing histone S activity in DCs, reducing intracellular MHCII antibody dimer levels, and suppressing lipopolysaccharide (LPS)-mediated surface expression of MHCII in DCs (Kitamura et al., 2005; Al-Kharboosh et al., 2020). Thus, inhibition of the STAT3 pathway can promote the maturation and aggregation of DCs by inhibiting IL-10, or by inhibiting the IL-6/STAT3 pathway. This contributes to maturation of DCs and activation of the subsequent immune response.

### 2.8 Effects of STAT3 activation on tumor-associated macrophages (TAMs)

TAMs play a key role in GBM angiogenesis, graded chain reaction of invasion, as well as invasion in the GBM microenvironment (Mantovani et al., 2017). Macrophages, produced from monocytes, are attracted to cytokines or chemokines secreted by glioblastoma cells, such as M-CSF, CCL family proteins, and CXCL family proteins. They are then recruited to GBM lesions, and are transformed into TAMs (Mantovani et al., 1992). TAMs are classified as M1-like macrophages and M2-like TAMs, and the roles of M1-like TAMs in the GBM microenvironment are mainly associated with the promotion of glycolytic metabolism and production of reactive oxygen species (ROS), which underlie their pro-inflammatory and cytocidal effects. In contrast, the main function of M2-like TAMs is to promote the repair of damaged tissues based on the bioenergy generated from oxidative metabolism (Locati et al., 2020). M1-like TAMs accumulated in the early stage of tumorigenesis inhibit the growth and division of GBM cells by producing a large number of cytotoxic factors such as NO, ROS, proinflammatory and cytokines. In addition, the TAMs could promote the necrosis of GBM cells, and further trigger the GBM cell apoptosis mediated by the immune system (Andersen et al., 2021). In contrast, a large number of aggregated M1-like TAMs contribute to chronic inflammation, thereby promoting the genomic instability of malignant cells, and eventually increasing the possibility of oncogene mutation and tumorigenesis (Liu et al., 2014). GBM cells usually induce the differentiation of TAMs to M2-like state during the pathogenesis of GBM. M2-like TAMs can produce a series of Th2 cytokines that promote GBM immune escape, together with cytokines that directly promote tumor cell proliferation such as IL-1β, TNF-α, and IL-6 (Vitale et al., 2019). STAT3 expressed in TAMs can suppress the antitumor immune response in the host, and promote the pathogenesis of GBM. Currently, it is believed that high expression of TAMs in GBM tissues is significantly related to the malignancy of GBM (Pyonteck et al., 2013). Activation of STAT3 in TAMs resulted in differentiation of immunosuppressive phenotype to M2 type, together with secretion of IL-10 and TGF-β1, which hampered its ability to mediate phagocytosis. These TAMs also showed a lack of molecules that were required to co-stimulate T cell activation and secretion of IL-23, which then induced the transformation of regulatory T cells (Tregs) into a more immunosuppressive phenotype (Sica et al., 2006).

### 2.9 Effects of STAT3 activation on tumor-associated Treg cells

The immune escape of GBM cells is closely associated with the regulatory roles of Treg cells (Amoozgar et al., 2021). Treg cells are recruited to the tumor foci by chemokines such as CCL22 in the tumor microenvironment, while CD4^+^ and CD25^+^ T cells are converted to Treg cells by specific cytokines (Yang et al., 2012a). The activation of STAT3 in the tumor-infiltrating Treg cells is significantly higher compared to Treg cells of normal cellular origin in the spleen (Dixon et al., 2021). The cytokines secreted by Treg cells such as TGF- α, and IL-10 could directly block CD8^+^ T cell activation or indirectly inhibit CD8^+^ T cell activation through DCs (Stewart and Trinchieri, 2009). Subsequently, this would cause the inactivation of APCs, together with suppressing the proliferation and antitumor activity of effector T cells including IFN-secreting Th1 cells and cytotoxic T lymphocytes. Finally, STAT3 enhanced the immune escape of cancer cells in the presence of IL-10 and TGF-α (Piersma et al., 2008). In a variety of cancer cells, administration of STAT3 inhibitor was reported to cause massive production of pro-inflammatory cytokines in the peripheral blood of immunocompromised GBM patients by inhibiting the STAT3 activity. Subsequently, it induced cytotoxic T cell proliferation and division, and suppressed aggregation of regulatory T cells, which then involved in preventing immune escape and suppressing glioblastoma growth and migration (Hussain et al., 2007; Ott et al., 2020).

### 2.10 STAT3 involved in the angiogenesis of GBM

Angiogenesis plays a decisive role in the growth, infiltration of GBM cells, and hypoxia tolerance is the initiating signal for angiogenesis and a key link in its late expansion (Cui et al., 2018). HIF-1, a dimer protein composed of HIF-1α and HIF-1β, plays an important role in microvascular events around the microenvironment and the progression of GBM (Barrientos et al., 2010). In cases of continuous tumor growth compressing the peripheral vasculature of GBM, it is not sufficient to meet the requirements for the oxygen and nutrient, and then the HIF-1 gene is heavily activated (Cui et al., 2018). The functional activation of HIF-α is not affected by regulatory effects in the hypoxia environment. After binding to the DNA hypoxia response element (HRE), HIF-1α promotes the cyclic activation of a series of downstream products, which then triggers the formation and bifurcation of GBM angiogenesis, and ultimately triggered the invasion of GBM cells (Xu et al., 2005). Antivascular therapy serves as an important treatment option for GBM. STAT3 is crucial to the basic expression of HIF-1, as well as its expression induced by upstream signal factors (Meng et al., 2018). PI3K Akt mediated HIF-1 and STAT3 activation is indispensable to *VEGF* gene transcription (Papadakis et al., 2010). Thus, STAT3 can not only directly regulate VEGF transcription by directly activating the *VEGF* promoter. In addition, it promoted VEGF expression through the PI3K/AKT/HIF-1 pathway, thereby promoting GBM vascular growth. Urokinase-type fibrinogen activator receptor (uPAR) and tissue proteinase B can interfere with the JAK-STAT pathway-dependent expression of VEGF, thereby inhibiting tumor-induced angiogenesis (Miyatake et al., 2013). However, due to the existence of the BBB, most VEGF inhibitors are not effective in treating GBM due to poor penetration (di Tomaso et al., 2011).

### 2.11 STAT3 participated in abnormal metabolism of GBM

In the presence of oxygen, GBM cells would preferentially initiate the glycolysis process, which triggers the generation of lactate (Duraj et al., 2021). This phenomenon is also called the Warburg effect featured by increased aerobic glycolysis and decreased mitochondrial function (Koppenol et al., 2011). This change is connected to the GBM cells’ quick production of macromolecules using lactic acid as a starting point. The proliferation and invasion of GBM cells required the energy and the macromolecular precursor that were closely related to the Warburg effect (Locasale and Cantley, 2010). The glucose is not fully utilized in the glycolysis process, and the number of ATP per mole of glucose produced by glycolysis is much less than that produced by aerobic respiration. However, glycolysis takes less time than the aerobic breathing (Koppenol et al., 2011). The activation of STAT3 is an important factor for the conversion of aerobic respiration to glycolysis. It induces aerobic respiration to glycolysis by promoting HIF-1 transcription and reducing the mitochondrial activity of glioblastoma cells. This metabolic pattern enhances the production of lactic acid and leads to the reduction of ROS production, which then protects the GBM cells from apoptosis and senescence (Avalle and Poli, 2018). Indeed, there is constitutive activation of STAT3 exists in GBM cells, and the Warburg effect shows significant increase. On this basis, we speculate that the Warburg effect in GBM cells is related to STAT3. Therefore, inhibition of STAT3 can reduce its survival and proliferation of GBM cells by modulating the glucose metabolism (Liu et al., 2013).

## 3 Relationship between radiotherapy and STAT3

Aberrant STAT3 activation is associated with radiation resistance in gliomas. Consistently, in patients with a high risk of recurrence after radiotherapy, there was pronounced STAT3 activation in gliomas (Zhang et al., 2011). As previously described, activation of STAT3 requires phosphorylation of its tyrosine (STAT3-Y705) residue and serine 727 (STAT3-S727) residue. As a protein kinase C inhibitor, Gö6976 can effectively improve radiosensitivity in gliomas. Additionally, it was merely downregulated in pSTAT3-Y705 negative conditions. This suggested that the radiosensitization of Gö6976 could be achieved through inhibiting the phosphorylation of STAT3-S727. A related hypothesis is that PSTAT3-S727 drives intrinsic radiation resistance, while PSTAT3-Y705 maintains resistance due to the interaction of tumor cells with their microenvironment (Ouédraogo et al., 2016). Consistently, resveratrol (3,4′,5-tri-hydroxy-trans-stilbene) was reported to effectively improve the radiosensitivity of glioma by inhibiting STAT3 activity. Specifically, it showed the ability to inhibit the expression of phosphorylated STAT3 and its downstream genes (e.g., Survivin, Cyclin D1, COX-2 and cMyc) in glioma cell lines (Yang et al., 2012b). Moreover, STAT3 enhanced the radiation resistance of GBM stem cells by regulating RCC2 to further activate the transcription of DNMT1 and enhancing the function of EZH2 in GBM. Furthermore, RCC2 can also release signals through STAT3 to activate transcription of DNA methyltransferase, resulting in hypermethylation of GBM suppressor genes and silence of related genes. This ultimately triggers the proliferation of glioma cells (Yu et al., 2019).

Radiation can significantly promote the phosphorylation of STAT3 Tyr705 in GBM cells by triggering EGFR phosphorylation and *IL-6* mRNA expression. Besides, the activation presents a dose-time dependence within a certain dose range (Ott et al., 2020).

Radiation therapy directly attacks cell DNA through radiation energy, resulting in single or double-strand breaks, generation of ROS, and apoptosis through oxidative damage (Liang et al., 2022b). It has been shown that GBM cells undergo a decrease in SOCS3 expression and an increase in FoxM1 expression after radiation. According to the previous description, SOCS3 downregulation contributes to the STAT3 activation, while FoxM1 protein can interact with STAT3 to increase the transcription of DNA repair-related genes (e.g., Mre11 and Rad51) (Lindemann et al., 2011; Liang et al., 2022b). After irradiation, STAT3/NF-κB and slug signaling pathways are also activated to further regulate intercellular adhesion molecule-1 (ICAM-1), which contributes to the invasiveness, interstitial migration, and activity (Kesanakurti et al., 2013).

Radiation has been reported to stimulate the granulocyte-macrophage colony-stimulating factor (GC-SF) within few hours, promoting the migration of bone marrow mesenchymal stem cells (MSCs) with a strong resistance to radiation. At the same time, recruited bone marrow MSCs are activated by the STING pathway through the pathway of C-C chemokine receptor type 2 (CCR2), which continuously increases the radiation resistance of GBM. The activation of T cells in the GBM microenvironment cannot be separated from arginine, and bone marrow MSCs can produce high levels of arginase 1 (Arg1), which inhibits the activation of T cells in the microenvironment by degrading arginine degradation. Subsequently, it could suppress the immune response and promote the progression of GBM (Moreira et al., 2021).

Radiotherapy can enhance their antigen presentation ability of DCs, and induce the production of cytotoxic T cells as well as its aggregation to the GBM cells. Irradiation stimulates the activation of a variety of signal pathways including MHC-I, TAA, and Fas/Fas ligand pathway, which is closely related to the increased susceptibility of GBM cells to cytotoxic T lymphocytes (Sefrin et al., 2019). Radiation-induced TME transformation is a “side effect” in which radiation induces activation of the interferon gene stimulating factor (STING) pathway in tumor-infiltrating DCs through activation of cGAMP synthases (CGAs). When cellular DNA is damaged, CGAs will be activated and involve in the catalysis of GMP and AMP in cytoplasmic DNA. The generated cGMP would activate the STING proteins, followed by activating downstream immune pathways and inducing cells to produce many transcription factors (e.g., NF-κB) (Kwon and Bakhoum, 2020). This will further activate STAT3 and the immune response against GBM. In addition, the enhanced immune response to GBM tissue mediated by radiation is related to the vascular cell adhesion molecule-1 (VCAM-1), and the expression of VCAM-1 on GBM endothelial cells is influenced by a variety of upstream effectors. After radiation exposure, the inflammatory cytokines such as IL-1β, tumor necrosis factor (TNF)-α, and type I and type II interferons are overexpressed in GBM cells, leading to upregulation of VCAM-1 expression (Corroyer-Dulmont et al., 2020), while the upregulation of intercellular adhesion molecule 1 (ICAM1) and VCAM1 in GBM blood vessels results in the wide infiltration of T lymphocytes in GBM tissues. Meanwhile, VCAM-1 and ICAM1 can induce migration of neutrophils to GBM through circulating tumor cells (CTC)-neutrophils and other forms. On this basis, the neutrophils can quickly penetrate the GBM microenvironment after radiation. Subsequently, it triggered the release of reactive oxygen species (ROS) and killing effects to the GBM cells (Kesanakurti et al., 2013; Lin et al., 2019).

Radiation could enhance the cytotoxicity of NK cells, and promote the aggregation of CD8^+^ cytotoxic T lymphocytes and M1 macrophages, while the M1 macrophage infiltration can promote inflammatory responses and inhibit GBM cell growth. Besides, radiation can also upregulate the expression of Fas and IFN-γ. Radiotherapy can play its immune-stimulating role and enhance the killing effects of the immune system on GBM cells by reducing the aggregation of infiltrating regulatory T cells (Treg) lymphocytes in tissues and inhibiting the PD-1/PDL-1 pathway (Portella and Scala, 2019).

**FIGURE 1:**
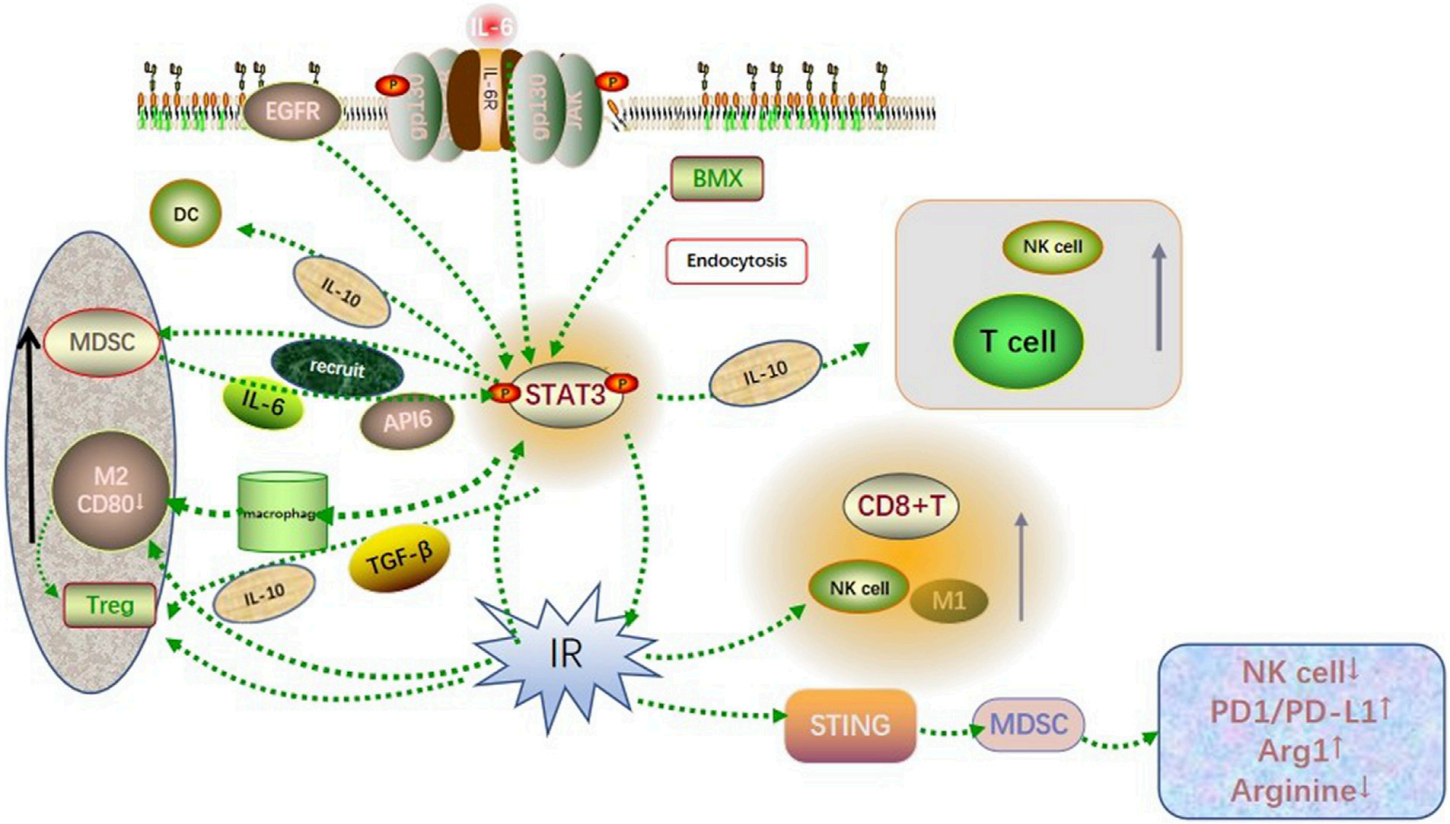
Co-influence of STAT3 and radiation on the GBM microenvironment. Activated STAT3 recruited immunosuppressive cells such as MDSC, M2-like TAM, and Treg to infiltrate the GBM microenvironment through cytokine signaling such as IL-6, IL-10, and TGF-β. Besides, it involved in recruiting immune cells such as T cells, DC cells, and NK cells to accumulate toward the tumor site. The radiation-induced DNA damage triggered the recruitment and activation of NK cells, M1-like TAM, CD8^+^ T cells, and other immune killer cells, while activation of the STING pathway leading to excessive activation of the PD1/PDL1 pathway and reduced arginine levels in the tumor microenvironment. Both showed synergistic and antagonistic effects on immunity, which finally resulted in the immunosuppression state in the GBM microenvironment.

Radiation has been shown to activate the GBM microenvironment immune system while simultaneously activate the immunosuppressive pathway, resulting in GBM resistance to radiotherapy. Overexpression of type I and type II interferons, together with significant infiltration of T lymphocytes, may promote PD-L1 upregulation in GBM cells exposed to the irradiation (Jarosz-Biej et al., 2019). The upregulation of PD-L1 in GBM cells can block the antitumor function of activated T cells and NK cells, hinder the recognition of GBM cells by immune surveillance. This contributes to the immune escape of GBM cells. In addition, radiation causes downregulation of co-stimulatory CD80 and CD86 molecules on immature DC cells and hinders T cell activation. On this basis, radiation induces apoptosis of GBM cells and emergence of a large number of tolerant DCs, inducing a suppressive Treg lymphocyte population (Jarosz-Biej et al., 2019). These suggest that radiotherapy, although it can kill tumor cells, creates synergistic effects with activated STAT3 in the tumor immune microenvironment, recruiting immunosuppressive cells and enhancing radiation resistance in gliomas (Figure 1).

Radiation has been shown to induce EMT and enhance the motility and invasiveness of gliomas, in which sublethal doses of radiation have been shown to enhance the migratory and invasive behavior of glioma cells (Wild-Bode et al., 2001). STAT3 is involved in IR-induced EMT and invasion through upregulation of molecules involved in EMT (e.g., N-calmodulin, wave protein and uPA), invasion (e.g., MMP-2 and MMP-9) and angiogenesis (e.g., VEGF and iNOS), respectively. Snail, a transcription factor riched in zinc finger structures, is a key transcription factor driving the EMT program. Radiation can increase FoxM1 expression by inducing STAT3, and FoxM1 binds directly to the Snail promoter to induce Snail expression and participate in the EMT process (Lee et al., 2017).

## 4 Targeting STAT3 is a new direction for treating GBM

### 4.1 Natural inhibitors of STAT3

Many natural substances have been reported to inhibit the STAT3 activity *in vitro*. For instance, resveratrol, a natural polyphenol with a stilbene structure derived from plants and fruits, has been shown to inhibit the proliferation and invasion of glioma cell in a STAT3-dependent manner. Resveratrol can penetrate the blood-brain barrier, and intrathecal injection allows high drug concentrations *in vivo* (Qian et al., 2022). Unexpectedly, it quickly metabolizes after systematical administration, resulting in very low bioavailability. To solve this problem, researchers increased the bioavailability of resveratrol by encapsulating it in transferrin-containing liposomes (Transferrin). GBM development was not significantly inhibited following intravenous treatment. Various natural STAT3 inhibitors, such as ursolic acid and the cryptotanshinone derivative KYZ3, have limited potency, unacceptable toxicity, quick metabolism, and/or poor blood brain barrier permeability, making them inappropriate for clinical application in the treatment of GBM (Jhaveri et al., 2018).

### 4.2 siRNAs targeting STAT3

RNA interference is an innovative approach to specifically target gene silencing, but in fact siRNA is not as effective as expected. siRNA molecules enter the body, circulate in the blood until they reach the tumor site and are finally carried into the tumor cells. Indeed, many difficulties prevent this goal, such as the presence of enzymes in the blood that may degrade siRNA, and blood-brain barrier, blood Tumor Barrier (BTB) and other barriers that prevent siRNAs from entering cancer cells (Mirzaei et al., 2021). Aptamers are small molecules of oligonucleotides capable of binding their targets with high affinity and specificity by obtaining structured folding. They hold great promise as antagonists of tumor-associated proteins and as secondary reagents for delivery carriers to target cells (Yoon et al., 2019; Catuogno et al., 2021). Camorani et al. obtained platelet-derived growth factor receptor β (PDGFRβ) aptamer, named Gint4.T, using the Cell-SELEX method. On this basis, the researchers further developed AsiC, a chimera targeting STAT3 siRNA combined with Gint4.T (Esposito et al., 2018). Related studies have shown that AsiC can effectively inhibit the multiplication and migration of glioblastoma cells and has high specificity with a stable blood concentration. Several miRNAs have been reported to be deregulated in GBM, governing different aspects of this tumour, including the maintenance and propagation of the GSCs. For instance, miR-10b acts as an oncomiR and is required for GSC self-renewal and proliferation, and the targeted delivery of a miR-10b antagonist reduces GSC propagation. GL21.T, an inhibitor ligand specific for receptor tyrosine kinase (RTK) Axl, is often used to target miR-10b, and we found that combined treatment with Gint4.T-STAT3 and GL21.T-anti-miR-10b complexes significantly abrogated the proliferation of GSCs. Here we found that the combined treatment of Gint4.T-STAT3 with GL21.T-10b resulted in a synergistic and drastic inhibition of GSC self-renewal (Esposito et al., 2020).

### 4.3 Exosomes-based strategies to target STAT3 for treating GBM

Exogenous An2-siRNA is an exosome that targets STAT3, a specific ligand for low-density lipoprotein receptor-related protein 1 (LRP-1), which is highly expressed on the surface of GBM cells and BBB endothelial cells. The researchers exploited the tumor homing properties of M1 macrophages to extract exosomes from them that readily aggregate toward tumors, and wrapped siRNA molecules in the inner lumen of exosomes by sonication to protect siRNA from degradation by ribonucleases and phagocytosis by macrophages, followed by An2 *via* 1,2-distearoyl-sn-glycero-3 phosphoethanolamine-N-(maleimide ((polyethylene glycol)-2000) (DSPE-PEG2000-MAL) that was attached to the exosome surface to form exo-An2-siRNA. By binding to exo-An2, siRNA is able to cross the BBB, relying on the specificity of An2-LRP1 binding accurately to the GBM. When reaching the GBM site, exo-An2-siRNA is taken up by GBM cells and the abundant siRNA molecules are released into the cytoplasm, thereby silencing the *STAT3* gene and inducing apoptosis in U87 MG cells. It was shown that exo-An2-siRNA avoided the rapid clearance of siRNA *in vivo*, which made siRNA more readily available to glioma cells. This effectively increased the efficiency of siRNA in silencing STAT3 and promoted apoptosis in glioma cells compared to free siRNA. In animal experiments, exo-An2 exhibited good BBB permeability and tumor aggregation in U87 MG transplanted tumors of tumor-bearing BALB/c nude mice. MST was the longest (24 days) in Exo-An2-siRNA-treated mice compared to controls, and Ki67 staining showed suppression of U87 MG cell growth in Exo-An2-siRNA-treated mice. This was attributed to good BBB permeability and accumulation of exogenous An2-siRNA in *situ* U87 MG xenograft tumors. In addition to siRNA, this vector could provide a pathway for other drugs targeting STAT3 to penetrate the BBB for the treatment of gliomas (Liang et al., 2022a).

### 4.4 Chemopreventive pharmacological approaches targeting GBM through diet-derived intervention on STAT3

TGF-β is a multifunctional cytokine that acts as a potential tumor suppressor in the early stages of tumorigenesis as a downstream signaling molecule through Smad and Smad non-dependent signaling pathways as a promoter of EMT and tumor metastasis. Among the specific glioma biomarkers that promote invasion and metastasis, membrane-1 matrix metalloproteinase (MT1-MMP) is a key membrane-bound matrix metalloproteinase involved in extracellular matrix (ECM) degradation. The established U87 grade IV human glioblastoma cell model showed an interaction between transforming growth factor-β signaling and MT1-matrix metalloproteinase. The overall phosphorylation status of Smad2/3 and STAT3 downstream of TGF-β was significantly reduced in cells silenced by MT1-MMPs. The diet-derived extract and epigallocatechin-3-gallate (EGCG) inhibit MT1-MMP-mediated downstream signaling involving STAT3. In glioma cell line U87 treated with 30 μm EGCG, it was observed that EGCG effectively reduced WT-MT1-MMP-mediated Src and STAT3 phosphorylation, upregulated glioma cell apoptosis and inhibited neurosphere formation in glioma cells (Djediai et al., 2021).

### 4.5 STAT3 inhibitor in combination with radiotherapy

The treatment options of GBM includes surgery, radiotherapy, temozolomide-based chemotherapy, and the combination of radiotherapy and temozolomide-based chemotherapy. Complete resection of GBM is still a challenge due to the invasive growth pattern of GBM cells. Besides, GBM patients are more likely to relapse due to the resistance to radiotherapy and chemotherapy. Current studies have shown that GBM stem cells are closely related to GBM cells resistance to radiotherapy and chemotherapy (Wang et al., 2021).

STAT3 inhibition can effectively inhibit the growth and migration of GBM stem cells and promote GBM cell apoptosis. Therefore, we believe that inhibition of STAT3 can effectively combat the chemoradiotherapy resistance of GBM stem cells and improve the therapeutic effect of GBM patients. A large number of literatures support that the activation of STAT3 is closely related to the drug resistance of targeted therapy. However, STAT3 inhibitors show no significant effects on the survival of tumor-bearing mice (Han et al., 2019). Due to the interaction of several signaling pathways that lead to “out of control” of GBM, including EGFR, PIK3CA, PDGF, and NF-κB, inhibition of at least one of these pathways is likely to lead to reactivity upregulation of other pathways (Pan et al., 2021), which may be largely responsible for the historical failure of targeted therapies.

STAT3 inhibition, or a combination of STAT3 inhibitor and radiotherapy, leads to immune reprogramming of TME in a mouse model of radioactive genetic immunity in GBM, which contributes to the interaction of DC cells and T cells, as well as antigen presentation. This suggests that modulating the therapeutic effects of STAT3 inhibition requires a full-functional immune response (Wang et al., 2019). The complex tumor microenvironment of GBM results in no significant improvement in patient survival as most of the patients only received administration of STAT3 inhibitors (Wang et al., 2020). Current studies have shown that the combination of whole-brain radiotherapy and WP1066 can effectively prolong the survival time of tumor-bearing mice from 23 days to 32 days. Magnetic resonance imaging (MRI) indicated less GBM cell survival in the mice received combined treatment, demonstrating that the combined treatment inhibited the growth of GBM cells. At the same time, immunohistochemical analysis showed that the degree of phosphorylated STAT3 staining was lighter in mice treated with whole-brain radiotherapy combined with WP1066 (Ott et al., 2020).

### 4.6 STAT3 inhibitor in combination with chemotherapy

The mechanism of temozolomide is the induction of DNA methylation of guanine at the O6 position, where O6-methylguanine mismatches with thymine and leads to the genomic double-strand breaks, as well as cell cycle arrest and apoptosis (Cheng et al., 2005). O6-methylguanine DNA methyltransferase (MGMT) involved in the demethylation of guanine at the O6 position, which is closely related to the temozolomide resistance. As previously described, upregulation of MGMT and STAT3 in GBM cell line U87 is accompanied by the acquisition of temozolomide resistance (Kohsaka et al., 2012). STAT3 inhibition enhanced the efficacy of TMZ by downregulating MGMT gene expression in TMZ-resistant GBM cell lines, while GBM with knockdown of STAT3 gene also exhibited enhanced sensitivity to temozolomide (Han et al., 2016).

STAT3 inhibitors can disrupt the BBB, allowing drugs that would otherwise be unable to penetrate the BBB. Ibrutinib can selectively disrupt the permeability of the BBB and enhanced the delivery of chemotherapies that with poor penetrating capacity to the BBB (e.g., etoposide), thereby prolonging survival time in mice (Zhou et al., 2017).

Anlotinib, a novel multi-targeted tyrosine kinase inhibitor used as an anti-angiogenic agent for treating a variety of tumors, is reported to inhibit angiogenesis in GBM by inhibiting the JAK2/STAT3/VEGFA signaling pathways and inducing autophagy in GBM cells by increasing Beclin-1 and microtubule-associated protein 1 light chain 3B (LC3B). The expression levels of Beclin-1 and microtubule-related protein 1 light chain 3B (LC3B) was increased to induce autophagy and apoptosis in GBM cells and to inhibit their invasion and metastasis. Temozolomide can effectively enhance the antitumor ability of anlotinib, and the combination of the two drugs inhibited JAK2/STAT3/VEGFA signaling more effectively than either drug alone under *in vitro* conditions (Xu et al., 2022).

### 4.7 STAT3 inhibitor in combination with immunotherapy

The hypoxic tumor microenvironment significantly enhances PD-L1 expression on MDSC through directly binding of HIF-1 to the transcriptionally active hypoxia response element (HRE) in the PD-L1 proximal promoter (Noman et al., 2014). Then the MDSC produces IFN-α, which activates Jak1/STAT1 signaling pathway and upregulates PD-L1 expression by binding to the type 1 interferon receptor (IFNAR1) (Xiao et al., 2018). Currently, blocking the MDSC recruitment to the tumor microenvironment could increase the susceptibility of cancer cells to anti-PD-L1 therapy (Highfill et al., 2014). Meanwhile, the secretion of IL-6, IL-10, and TGF by MDSC showed obvious decrease under hypoxic conditions in the patients received anti-PD-L1 treatment, and MDSC exhibited a diminished ability to suppress T cells. STAT3 activation can promote the accumulation and aggregation of MDSCs, and its inhibition can suppress the level of MDSC in the tumor microenvironment and improve the therapeutic effects of anti-PD-L1. Therefore, STAT3 inhibitor-targeted therapy combined with anti-PD-L1 immunotherapy may become a new direction for treating GBM (Noman et al., 2014).

### 4.8 STAT3 inhibitor combined with anti-vascular therapy for treating GBM

Anti-angiogenic therapy is one of the current treatments for glioma. In the presence of abnormal neovascularization in GBM cells, anti-angiogenic therapy can effectively reduce tumor angiogenesis by targeting VEGF, serving as a key regulatory molecule in the angiogenesis. Thus, it could inhibit the tumor growth and angiogenic brain edema. The mean percentage of p-STAT3-expressing cells showed significant increase in tumors that failed to receive VEGF inhibitors treatment compared to samples from patients receive no non-anti-angiogenic containing therapy (Fang et al., 2021). However, the delivery of VEGF inhibitors to the target sites across the BBB is still a challenge. In addition, the upregulation of p-STAT3 would affect the treatment efficiency of VEGF-targeted anti-angiogenic agent, as anti-angiogenic therapy-mediated induction of hypoxia in the glioblastoma microenvironment can be directly activated by STAT3/HIF-1/VEGF that was associated with tumorigenesis and progression (Yuan et al., 2015). In addition, the combination of STAT3 inhibitor (i.e., AZD1480) and VEGF inhibitor (i.e., cediranib) significantly reduced GBM volume and microvessel density in murine xenograft models of GBM (de Groot et al., 2012). All these suggested that upregulation of the STAT3 pathway can mediate resistance of GBM cells to anti-angiogenic therapy, while the combination of STAT3 inhibitor and anti-vascular therapy inhibitor can delay or reduce the resistance to the anti-angiogenic therapy.

## 5 Research status of STAT3 inhibitors

**FIGURE 2:**
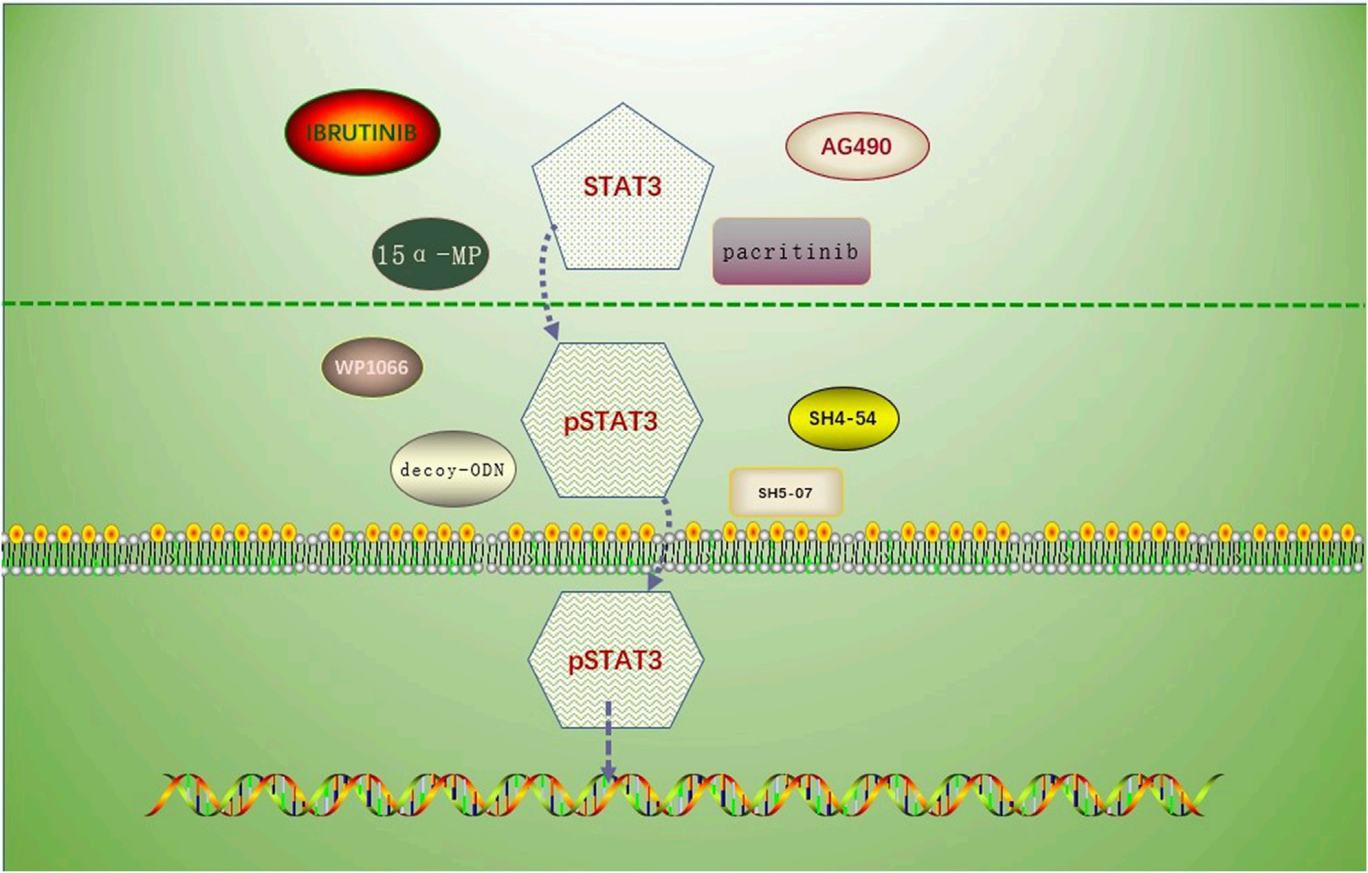
Drug mechanism involving the prevention of STAT3 phosphorylation or inhibition of p-STAT3 nuclear translocation. Drugs associated with the inhibition of STAT3 phosphorylation: AG490, MNS1 Leu and MNS1 MV, pacritinib, 15 α- MP; drugs associated with the inhibition of STAT3 nuclear translocation: SH5-07 and SH4-54, and decoy-ODN.

This article introduces a number of STAT3 inhibitors with quick research advancements (Table 1), including pacitinib, 15-MP, mns1 Leu and mns1 MV, WP1066, and AG490, *etc.* Clinical trials for these medications have not yet demonstrated their viability in people, and more clinical study is anticipated (Figure 2).

**TABLE 1:** Studies on the inhibitory effect of various STAT3 inhibitors on STAT3 pathway.

Drug	Experimental design	Whether it can cross the blood-brain barrier	Reference or clinical trials ID
WP1066	Phase-I	Yes	NCT01904123
SH5-07	Animal experiment: mice	No	Yue et al. (2016)
SH4-54			
decoy-ODN	Cell experiment: U87 MG	No	Gharbavi et al. (2020)
AG490	Cell experiment: U87 MG	No	Zheng et al. (2014)
MNS1-LEU	Animal experiment: mice	Yes	Zhang et al. (2019)
MNS1-MV			
Pacritinib	Animal experiment: mice	Yes	Chuang et al. (2019)
15α-MP	Animal experiment: mice	No	Hilliard et al. (2017)
Ibrutinib	Phase-I	Yes	NCT03535350

### 5.1 Targeted blocking STAT3

Aberrant activation of STAT3 in GBM cells inhibited the production of pro-inflammatory cytokines that involved in the maturation of DC. Thus, there is usually a deficiency of DC-dependent T cells in GBM cells (Ott et al., 2020). As a caffeic acid analog with the characteristics of penetrating BBB, WP1066 could inhibit the activation of p-STAT3 protein mainly through blocking the nuclear translocation of p-STAT3 protein, preventing the p-STAT3 protein from binding to DNA, and relieving STAT3 from restricting the secretion of pro-inflammatory factors. This promoted the proliferation of dendritic cell-dependent T cells and enhanced immune competence (Hussain et al., 2007). High-energy radiation could affect the DNA integrity in GBM cells and the phosphorylation of STAT3. Additionally, the radiation promoted the nuclear localization of STAT3, and inhibited the immune effect of immune cells. Currently, WP1066 combined with whole-brain radiotherapy was superior in up-regulating the expression of GBM microcirculation antigen and T cell activation, as well as inducing immune memory. These indicated that the combination of targeted STAT3 treatment and radiotherapy could effectively prolong the median survival time of experimental animals (Ott et al., 2020).

STAT3 inhibitors (e.g., SH5-07 and SH4-54) are hydroxamic and benzoic acid analogues, both of which could interrupt the binding of activated STAT3 to nuclear DNA, providing preclinical evidence for the application of SH5-07 and SH4-54. At the same time, SH5-07 and SH4-54 are highly specific for STAT3, without affecting the function of STAT3-independent genes (Yue et al., 2016).

Antisense oligonucleotides are a class of molecular drugs that inhibit the expression of specific genes by sequence-specific binding to target DNA or mRNA. Antisense oligonucleotide STAT3 transfected into U251 cells can inhibit the expression of STAT3, reduce the content of STAT3, and inhibit the proliferation and invasion of glioblastoma cells. Under *in vitro* conditions, blocking of STAT3 signaling pathway using double-stranded decoy oligodeoxynucleotides (ODNs) can inhibit the growth of GBM by suppressing the expression of target genes at the downstream of pSTAT3 (Gu et al., 2008). *In vivo*, decoy-ODN could downregulate the target genes of STAT3 at transcriptional and translational levels, which then inhibited the proliferation and promoted apoptosis in xenografts (Shen et al., 2009).

### 5.2 Targeted inhibition of STAT3 activation

As a selective JAK2 inhibitor, AG490 was the inspiration for the synthesis of WP1066. In GBM cells, AG490 suppressed STAT3 activity by decreasing the phosphorylation of STAT3-Tyr705, MMP-2 expression, and the activity of associated enzymes. Besides, it has been shown to decrease migration and invasion of GBM cells *in vitro* (Zhang et al., 2018a).

MNS1-LEU and MNS1-MV are newly synthesized pyrazole derivatives that impede pSTAT3 translocation to the nucleus and nuclear DNA binding. MNS1-LEU and MNS1-MV could enhance the apoptosis and prevented GBM cell migration in cellular assays. Whereas, in animal experiments, MNS1-LEU and MNS1-MV could penetrate the BBB. In addition, both MNS1-LEU and MNS1-MV were stable with no toxicity to the tissues as they were stable in human plasma after 96 h at 37°C (Zhang et al., 2019). Pacritinib is a new compound targeting JAK2. *In vitro* studies indicated that pacritinib inhibited JAK2 and improved the response to temozolomide in temozolomide-resistant GSC. *In vivo* studies have also shown that pacritinib can cross the blood-brain barrier (Patel and Odenike, 2020). 15α-methoxypupehenol (15α-MP) was extracted from the Hyrtios sponge. Because 15α-MP inhibits pSTAT3-Tyr705 in human glioblastoma cells and recent animal tests support its anticancer effect against glioblastoma, activated STAT3 may be an antitumor target of 15α-MP (Hilliard et al., 2017). BMX is a non-receptor tyrosine kinase, belonging to the Tec kinase family, which acts through an SH2 structural domain to efficiently bind tyrosine to phosphorylated proteins and activate STAT3 (Guryanova et al., 2011). The unrestricted self-division ability and differentiation characteristics of glioblastoma stem cells are inseparable from BMX-mediated signal transduction. The results showed that BMX gene knockout could effectively inhibit the activation of STAT3 (Shi et al., 2018). Ibrutinib is a BMX inhibitor that crosses the blood-brain barrier and effectively inhibits the activation of glioblastoma stem cells STAT3. Current cellular and clinical data suggest that Ibrutinib significantly inhibits GSC-driven tumor growth and may improve survival in patients with glioblastoma. Indeed, some other inhibitors of the IL-6/JAK/STAT3 pathway, such as AZD1480, LLL12, OPB-31121, SH-4–54, are still under the investigation for the efficiency in treating GBM.

## 6 Conclusion

Rational treatment strategies need to take into account not only anatomical barriers such as the BBB, but also the tremendous heterogeneity within and between tumors. As preclinical studies have shown, JAK/STAT signaling is highly complex, and although, on balance, constitutive activation tends to promote tumor proliferation, angiogenesis, and immune escape, targeting upstream or downstream effectors is not always predictable. WP1066, the most rapidly investigated STAT3 inhibitor, can inhibit STAT3 phosphorylation by blocking GBM cell proliferation and induction of apoptosis in GBM cells (Iwamaru et al., 2007; Guo et al., 2012), and by inhibiting STAT3 can significantly improve the sensitivity of GBM to radiotherapy. The combination of STAT3 inhibitors with radiotherapy and chemotherapy may play an important role in the treatment of GBM in the future. However, human studies are still required to further illustrate the potential mechanisms. Because strategies based on the vulnerability of one or two key molecules are not likely to be generalized to the entire patient population, continued efforts on screening and validating biomarkers to stratify patients suitable for JAK/STAT combination therapy are critical to improving our understanding of GBM. Phosphorylated STAT3 also has significant implications for guiding glioma treatment. Recent data suggest that PSTAT3-S727 rather than PSTAT3-Y705 is a constant pathological feature of activated STAT3, which is involved in the resistance of GBM to gold standard therapies. Inhibition of PSTAT3-Y705 alone does not improve glioma resistance to existing therapies, and combined inhibition of PSTAT3-S727 and PSTAT3-Y705 downregulates pathological activation of STAT3 in glioma cells, glioma stem cells, and mesenchymal cells (Ouédraogo et al., 2016). Therefore, there is a need to identify and validate new STAT3 inhibitors for preclinical and/or clinical testing of two phosphorylated broad inhibitors.
